# Influence of Flux and Related Factors on Intermetallic Layer Growth within SAC305 Solder Joints

**DOI:** 10.3390/ma14247909

**Published:** 2021-12-20

**Authors:** Karel Dušek, Petr Veselý, David Bušek, Adam Petráč, Attila Géczy, Balázs Illés, Oliver Krammer

**Affiliations:** 1 Department of Electrotechnology, Faculty of Electrical Engineering, Czech Technical University in Prague, 16627 Prague, Czech Republic; veselp13@fel.cvut.cz (P.V.); busekd1@fel.cvut.cz (D.B.); petrac.adam@gmail.com (A.P.); 2 Department of Electronics Technology, Budapest University of Technology and Economics, 1111 Budapest, Hungary; gattila@ett.bme.hu (A.G.); billes@ett.bme.hu (B.I.); krammer@ett.bme.hu (O.K.)

**Keywords:** solder flux, reflow soldering, SAC solder alloy, solder mask, surface finish

## Abstract

Flux contained in solder paste significantly affects the process of solder joint creation during reflow soldering, including the creation of an intermetallic layer (IML). This work investigates the dependence of intermetallic layer thickness on ROL0/ROL1 flux classification, glossy or matt solder mask, and OSP/HASL/ENIG soldering pad surface finish. Two original SAC305 solder pastes differing only in the used flux were chosen for the experiment. The influence of multiple reflows was also observed. The intermetallic layer thicknesses were obtained by the image analysis of micro-section images. The flux type proved to have a significant impact on the intermetallic layer thickness. The solder paste with ROL1 caused an increase in IML thickness by up to 40% in comparison to an identical paste with ROL0 flux. Furthermore, doubling the roughness of the solder mask has increased the resulting IML thickness by 37% at HASL surface finish and by an average of 22%.

## 1. Introduction

Soldering technology is a predominant technique for joining electronic components on the printed circuit board (PCB). The tin-based solders with the help of fluxes are used for joining metal parts. The soldering process is a complex one, where a combination of flux, temperature, and materials all have a decisive influence on the resulting joint. During the soldering process, flux reduces the oxides present on the metal surfaces and prepares the conditions for good wetting by solder. The formation of the intermetallic layer (IML) is inevitable and begins when the solder starts to wet the substrate. According to the work by Tu et al. [1], a thin IML ensures a good metallurgical bond between the solder and metal parts. Furthermore, IML improves the thermal properties and is beneficial to wettability [2]. On the contrary, excessive formation of IML at the solder/substrate interface can weaken the mechanical properties of the solder joint due to the brittle nature of the IML and different coefficients of thermal expansion to the PCB and solder [3]. Therefore, the understanding of intermetallic compound (IMC) formation during the soldering process and its further growth during equipment usage is very important. The main factors influencing the IML thickness (based on the available literature) are described in the following section. However, to the best of our knowledge, no comprehensive study dealing with the influence of the flux type and specifically with its amount on the IML growth, which is the main goal of this article, has been published to date. In this paper, another goal was to find the factors that influence the IML thickness the most upon entering the soldering process, and thus the solder joint reliability.

### 1.1. Material Effect on IMC Growth

The widely used material of soldering pads in electronic production is copper (Cu), due to its good solderability and high electrical and thermal conductivity [4]. Sn-based solders at Cu substrate are characterized by the presence of Cu_3_Sn (ε) and Cu_6_Sn_5_ (η) intermetallic layers that are formed at the solder-substrate interface [3]. As per the restriction of hazardous substances (ROHS) directive, one of the widely used solders are of the SAC type (SnAgCu). Therefore, Ag_3_Sn IMCs are also present in Sn-based solder joints [5,6,7].

**Figure 1:**
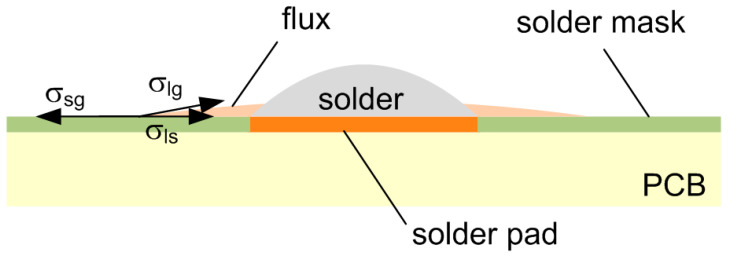
Surface tension equilibrium diagram, where σ_sg_ (Nm^−1^) is the surface tension between solid and gas, σ_ls_ (Nm^−1^) is the surface tension between liquid and solid, σ_lg_ (Nm^−1^) is the surface tension between liquid and gas.

Another common metal that is used in surface finishes on copper pads is nickel (Ni), which is used, for example, as an intermediate layer under gold surface finishes. It has good wettability, but has a lower reaction rate with Sn-based solders in contrast to copper [8]. The formation of Ni_3_Sn_4_ intermetallic layer is characteristic for the interface between the Sn-based solder and nickel [9], and its growth rate is slower. Therefore, nickel can serve as an excellent reaction barrier that restricts the excessive dissolution of Cu into the solder joints [10,11]. The structure of the solder pad (such as its roughness) also influences the IMC, though not primarily in its growth rate, but rather in the IMC ratio between various intermetallic phases, such as Ag_3_Sn, Cu_3_Sn, and Cu_6_Sn_5_ phases [12], where their presence is both inevitable and necessary. On the contrary, it may cause reliability issues [5]. As already indicated, the formation and growth of intermetallic layers depend on the solder alloy composition [13,14]. In addition, it depends on the flux type [15] since the flux influences the wetting of the solder, due to the oxide reduction on soldered surfaces and solder paste particles. This interaction can change the diffusion process during IML formation. The flux activation occurs during its heating, the viscosity decreases, and the spreading to the surrounding of the soldering pads appears. The spreading is influenced by the solder pad type (solder mask defined/copper pad defined) and solder mask surface parameters, specifically solder mask roughness [16,17]. The flux spread depends on surface tension vectors. The surface tension relationship to the spread area on the solder mask, including a detailed vector description and mutual relation is explained in reference [18] in detail and is shown in Figure 1.

The smaller the surface tension σ_ls_, the better the wetting conditions (larger spread) of a liquid. Therefore, the higher spread of the flux lowers the amount of the flux that remains in the joint, and may thus, have an effect on the creation of the IML.

### 1.2. Intermetallic Layer Growth

The interfacial reaction between solder alloys based on Sn and the soldering substrate can be divided into two stages: The formation of the intermetallic layer during the soldering process and the growth of the intermetallic layer at a later time, during equipment usage or thermal aging [19]. The sooner the solder starts to wet the surface, the sooner the formation of IMC starts.

The interfacial reaction between the solder and soldered substrate can be described by Fick’s diffusion law and simplified as:

(1) $$L=\sqrt{D\times t}$$

where *L* is the average thickness of the IMC layer, *D* represents the diffusion coefficient, and *t* is time [20].

However, this equation is valid only at the start of the wetting process and cannot be used to describe the growth of the IML thickness during longer reflow times, where it approaches a rather parabolic growth curve [3].

The intermetallic layer thickness increases with the increased aging temperature and aging time [2]. An increase in IML thickness has also been observed after increasing the reflow temperature (peak temperature), increasing the reflow time (time above liquidus) [21] or the number of reflow cycles. A parameter known as a heating factor (defined as the integral of the measured temperature over the dwell time above liquidus) has been used in several studies [22,23,24] to quantitatively describe the effect of the heating process (effect of the combination of temperature and time during reflow soldering) on the resulting thicknesses of intermetallic layers. However, the heating factor does not always play the decisive role, since it describes only the reflow phase of the soldering process. The IMC thickness is also influenced by the preheat phase, when the flux is activated, as shown in our previous work [25], by the direction of the heat flow [26] and also by the post reflow cooling rate [27].

The resulting IML is one of the quality and reliability parameters of the solder joints.

## 2. Materials

**Figure 2:**
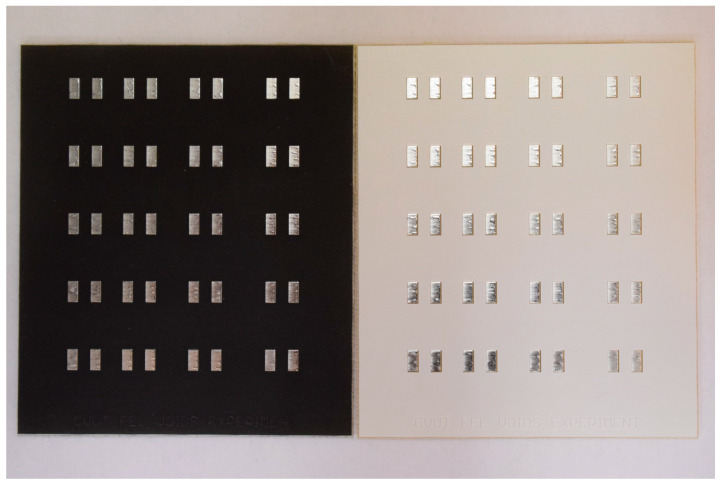
Design of PCB test board. Black solder mask with HASL surface finish (**left**); white solder mask with HASL surface finish (**right**).

For the experiment, a testing PCB with 40 copper-defined soldering pads with dimensions of 1.78 mm × 3.5 mm (see Figure 2) was designed. Three types of surface finish of the copper (organic solderability preservative (OSP), hot-air solder leveling (HASL), and electroless nickel-immersion gold (ENIG)), as well as two types of solder mask (white and black) were used. The solder masks differed in surface roughness.

Two types of SAC305 solder paste with the Sn97.5/Ag3/Cu0.5 solder alloy that differed only in the type of flux chemistry (ROL0 and ROL1, according to the IPC J-STD-004 standard) were used. The ROL0 flux contained less than 0.05% of halides, while ROL1 contained a 10 times higher content of halides, specifically less than 0.5% of halides. Both pastes were supplied by Shenmao Company, TaoYuan, Taiwan.

## 3. Experimental Setup

**Figure 3:**
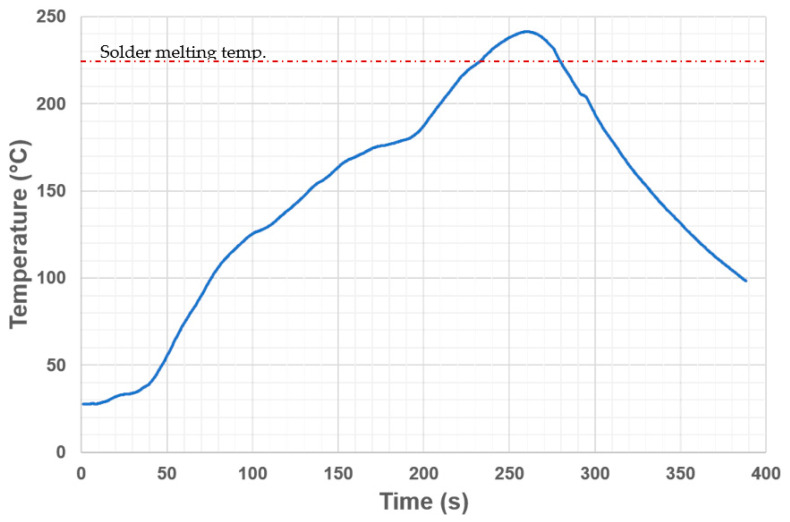
The temperature profile of the reflow oven used for sample preparation.

The solder paste was deposited on the pads by the stencil printer SAB 06 (ELPRO, Košice, Slovakia), using a steel, 100 µm thick stencil. The alignment error was below 0.1 mm. The solder paste was reflowed in a convection oven Mistral 260 (Spidé, Harderwijk, Netherlands) with three adjustable temperature zones (two for the preheating phase, the last one for the reflow phase). The temperature profile of the soldering process was measured by the KIC 2000 thermal profiler (KIC, San Diego, CA, USA) with a K-type thermocouple attached to the soldering pad. The reflow profile is shown in Figure 3. Half of the samples (12 boards) were reflowed twice to simulate the assembly of double-sided boards.

Overall, 24 different combinations of the solder mask, surface finish, flux, and the number of reflows were studied in our experiment. The four-letter marking of the samples is as follows: The first letter denotes the roughness of the solder mask (**B**lack/**W**hite), the second denotes the surface finish type (**O**SP, **H**ASL, **E**NIG), the third letter describes the flux activity type (ROL**0**, ROL**1**), and the last position denotes the number of reflow cycles (**1** or **2** cycles). Therefore, W-O-0-1 represents a sample with a white mask, OSP surface finish, solder paste with ROL0 flux, and one reflow cycle.

**Figure 4:**
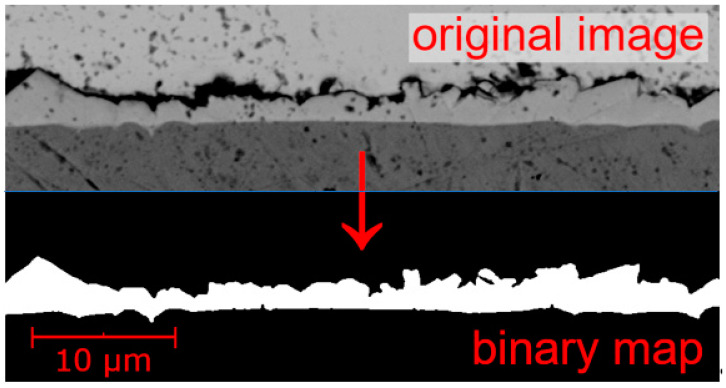
Conversion of the original IML SEM image to a binary map.

After the soldering process, we carried out the cross-sectioning of solder joints. The cross-sections were analyzed with the Phenom ProX scanning electron microscope (Thermo Fisher Scientific, Waltham, MA, USA). In total, 10 images with 10,000 magnification of the intermetallic layer area were taken for each combination of the solder mask, flux, surface finish, and the number of reflows. The images were further processed and converted to a binary map by the NIS Elements software (version 3.10, Laboratory Imaging s.r.o., Praha, Czech Republic)—the area of the intermetallic layer was selected (see Figure 4). With the use of a MATLAB script, the average number of white pixels in one column throughout the whole image was computed and converted to a real distance in µm. This value equals the average thickness of the intermetallic layer. The script also calculated a median thickness and a thickness variation for each analyzed SEM image. Although the median value might be sometimes better as it filters out extreme values, such as variations given by local IMC pervasions deep into the solder alloy volume, only the average thickness value is further presented in the graphs, in order to be consistent with other research works dealing with the IMC thickness.

In order to better understand the flux behavior during soldering, a complementary analysis of the flux spreading was conducted using the VK-X1000 confocal microscope (Keyence, Osaka, Japan). By scanning the area around the soldering pad, we obtained the total area and volume of the spread flux for each combination of solder mask and flux type.

## 4. Results

**Figure 5:**
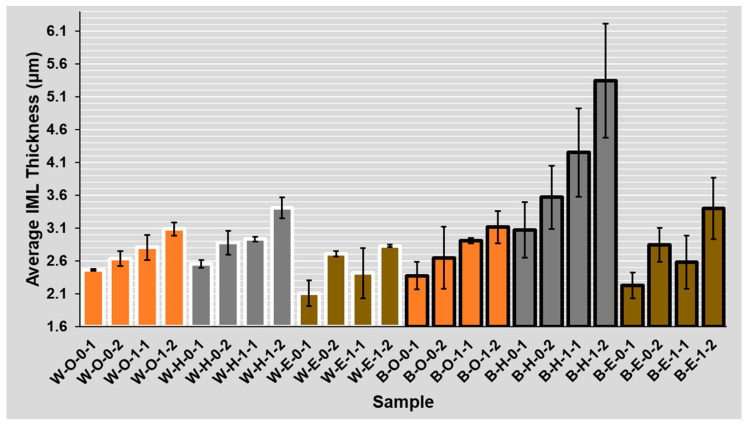
Average IML thickness for various combinations (**B**lack/**W**hite)—(**O**SP, **H**ASL, **E**NIG)—(ROL**0**, ROL**1**)—(**1**, **2** reflows).

The IML thickness evaluation was conducted for 24 different combinations of two flux types, three surface finishes, two solder masks, and the number of reflow cycles (see Figure 5). The average and median thickness in µm and average variation in percent were computed from the obtained SEM images. The variation gives information on thickness homogeneity. On average, it was 24%, and it did not vary between the observed combinations significantly.

### 4.1. Solder Mask Roughness Measurement

**Figure 6:**
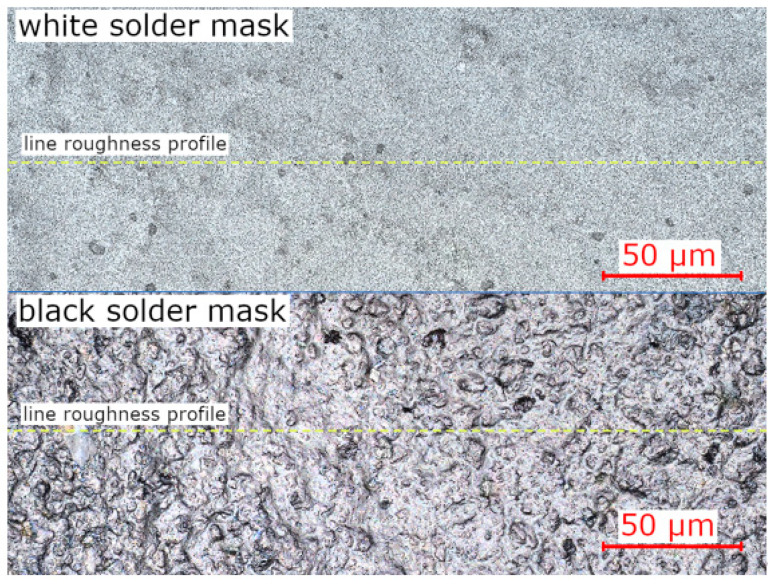
Solder masks—images from the laser confocal microscope. White-low roughness (**top**) and black-high roughness (**bottom**).

The measurement of the line (R_a_) and the surface (S_a_) roughness was used to determine the difference between the two selected solder masks. The measurement was conducted on the VK-X1000 laser confocal microscope and was repeated in 10 different areas for each solder mask type. Measuring the line roughness in perpendicular directions ensured that the obtained value did not depend on the manufacturing process. The black solder mask has an approximately two times higher line and surface roughness than the white mask (see Table 1). Moreover, the difference can be seen under the microscope (see Figure 6).

### 4.2. Flux Spreading Analysis

The Keyence VK-X1000 confocal microscope was used to measure the area of spread flux around the pad. The MultiAnalyzer software (version 2.1.3.89) from Keyence was used on the combined optical and laser image of the solder pad and its surrounding. The area of the flux and solder pad was marked automatically based on the Z-axis data above a threshold given by the solder mask plane. Then, a built-in smoothing algorithm and some manual corrections were performed in order not to mistakenly include the selected areas with protrusions. Finally, the solder pad area (6.23 mm^2^) was manually excluded from the selection. Then, the software automatically computed the area and volume of the selected part of the image.

**Figure 7:**
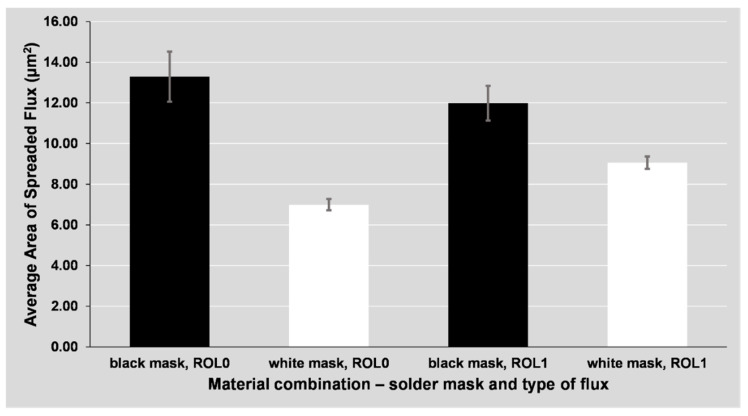
Average area of spread flux around the soldering pad depending on the solder mask and flux type.

**Figure 8:**
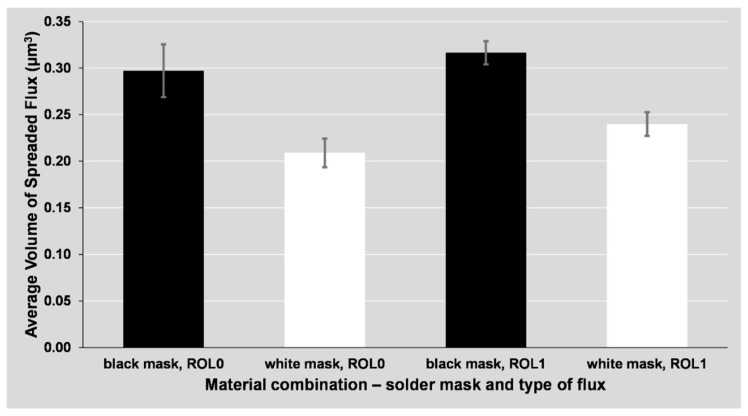
The average volume of spread flux around the soldering pad depending on the solder mask and flux type.

The differences in the area and volume of the spread flux are shown in Figure 7 and Figure 8. The columns represent the combined results of all surface finishes (ENIG, HASL, OSP), as the difference in spreading between the mentioned surface finishes is minor. The spreading depends mainly on the used flux and mask type. The use of glossy (white) mask led to a significant decrease of the spread area.

**Figure 9:**
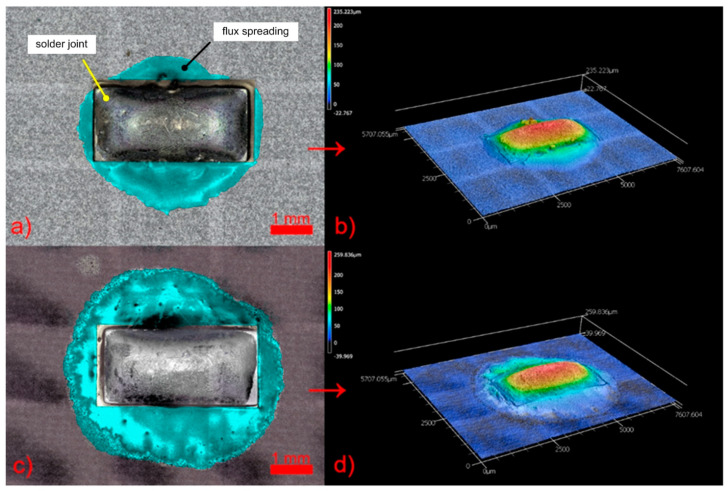
Analysis of flux spreading by the 3D laser confocal microscope. Solder joint on glossy (white) mask (**a**) and its 3D scan (**b**); solder joint on matt (black) mask (**c**) and its 3D scan (**d**). The volume of the flux was measured only in the highlighted area (cyan color)—shown in (**a**) and (**c**).

The slight differences between the graphs in Figure 8 and Figure 9 are given by the uneven distribution of the flux volume. Only the volume of the flux that was spread on the solder mask was evaluated (see the highlighted flux area in Figure 9a,b).

Figure 9 presents an example of the different spreading on both solder mask types obtained using a 3D confocal microscope.

### 4.3. Evaluation of Solder Mask Influence (**B**lack/**W**hite)

**Figure 10:**
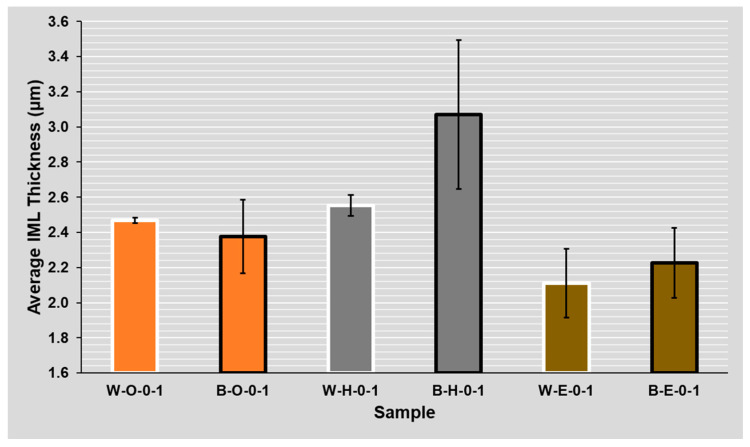
Solder mask influence on the average IML thickness for samples with the ROL0 flux (**B**lack/**W**hite)—(**O**SP, **H**ASL, **E**NIG)—(ROL**0**, ROL**1**)—(**1**, **2** reflows).

**Figure 11:**
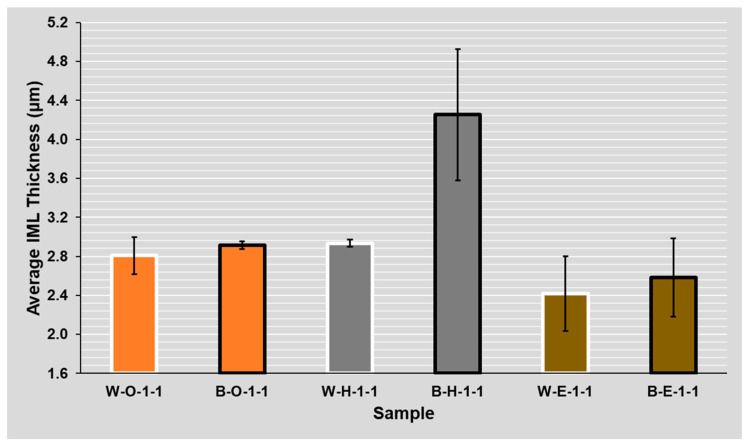
Solder mask influence on the average IML thickness for samples with the ROL1 flux (**B**lack/**W**hite)—(**O**SP, **H**ASL, **E**NIG)—(ROL**0**, ROL**1**)—(**1**, **2** reflows).

The apparent difference in IML thickness between the two masks can be seen on the samples with the HASL surface finish, where the increase of IML thickness on the sample with the black (double roughness) mask was about 37% on average (see Figure 10 and Figure 11). A significant difference was observed for ROL1 flux, where the IML was 22% thicker on average on the sample with the black mask compared to the sample with the white mask. For clarity, the results are shown for ROL0 and ROL1 fluxes separately and the twice reflowed samples were excluded.

### 4.4. Evaluation of Surface Finish Influence (**O**SP, **H**ASL, **E**NIG)

The thinnest IML exists with the ENIG surface finish, the thicker IML was observed with the OSP surface finish, and the thickest Cu-Sn IML was present on HASL, since the copper-solder IML interface is already a part of the HASL finish due to the manufacturing process of this surface finish. On the other hand, the OSP surface finish has the lowest increase of intermetallic layer between the first and second reflow (see Figure 5).

### 4.5. Evaluation of Flux Influence (ROL**0**, ROL**1**)

**Figure 12:**
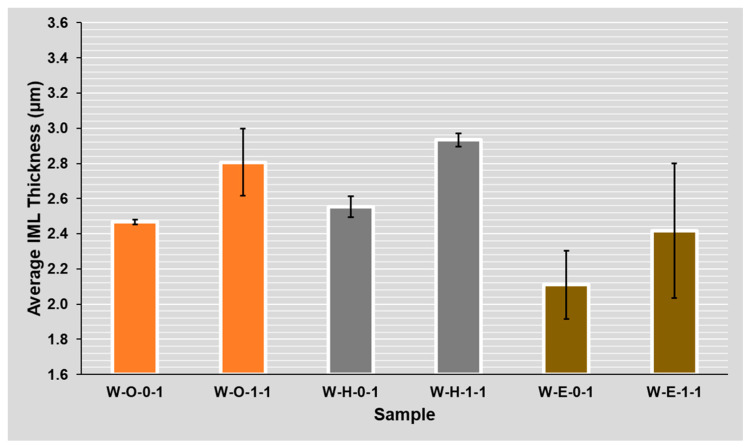
Flux type influence on the average IML thickness for samples with the **W**hite solder mask (**B**lack/**W**hite)—(**O**SP, **H**ASL, **E**NIG)—(ROL**0**, ROL**1**)—(**1**, **2** reflows).

**Figure 13:**
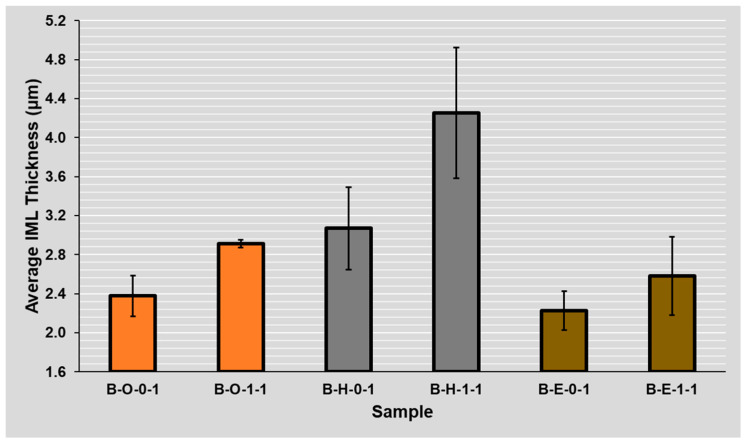
Flux type influence on the average IML thickness for samples with the **B**lack solder mask (**B**lack/**W**hite)—(**O**SP, **H**ASL, **E**NIG)—(ROL**0**, ROL**1**)—(**1**, **2** reflows).

Regarding the ROL1 flux, the intermetallic layer was up to 40% (1 µm) thicker compared to the ROL0 flux. The effect of the higher halide content within the flux on the IML thickness was significant on samples regardless of their mask type (glossy white mask—see Figure 12 and matte black mask—see Figure 13). A significant influence of the flux type on the intermetallic layer thickness is also visible when the results after the first and second reflow are compared. In the case of ROL1, the increase of intermetallic layer between the first and second reflow is higher when compared to ROL0. This is most remarkable in the case of HASL surface finish.

### 4.6. Influence of Several Reflows

The results clearly indicate the greater resulting thickness after the secondary reflow for all of the surface finishes (see Figure 5). This is in full agreement with the research works on this topic [28,29,30]. From the overall point of view, the highest increase in the thickness of the intermetallic layer is evident in the HASL surface finish and the lowest in the OSP surface finish. The highest intermetallic layer increase after the second reflow was observed at a combination of the black mask, HASL surface finish, and ROL1 type flux.

## 5. Discussion

The absolute IML thickness varied between two and four micrometers between the experiments, which is in line with other works studying IML properties, such as [14] or [31]. In this article, one of the main topics was to study the effect of the solder mask type (which causes a different amount of flux on the soldering pad, respectively, in the solder joint) on the IML thickness. The work by Piotrowska [32], which describes the liquid behavior on different surface morphologies, led us to this study. We used similar principles and built on different flux spreading on solder masks with different roughness [18]. The use of a glossy (white) mask led to a significant decrease in the spread area. Therefore, the flux was not drained away from the joint, and a higher amount of flux influences the solder joint formation during the soldering process. According to the obtained results (see Figure 5), higher amounts of flux present in the joint during its formation (on the white mask with low roughness) slow down the diffusion process and dampen the IML growth. This leads to a hypothesis that the flux layer—in addition to its useful function as an oxide reducing agent—can act as a diffusion barrier. Moreover, a hypothesis exists that the larger spread of the solder flux helps in washing away the oxide reduction residues and thus the wetting may start earlier, resulting in a thicker IML.

As stated in the results, the thinnest IML exists with the ENIG surface finish, where the nickel layer provides a diffusion barrier that inhibits the growth of Cu-Sn intermetallic. This observation agrees with [33], where the property of Ni_3_Sn_4_ intermetallic is its significantly slower growth. On the contrary, the thickest layer was observed at HASL surface finish. The reason is that the IML was already created at the soldering pads before our experiment due to the nature of the HASL surface finish manufacturing process.

The flux halide content played a significant role in the effect on IML thickness. ROL1 flux is, due to the higher content of halides [34], more “aggressive” and this was clearly visible in the results (see Figure 12 and Figure 13). The assumption is that the higher activity allows faster wetting, the diffusion thus started earlier, and the IML had more time to grow.

The flux creates different diffusion conditions that affect the further IML growth with ENIG and HASL, while the OSP behaves initially as a diffusion barrier. This is evident not only from the IML thickness measured after the first reflow, but also after the second reflow.

The hypotheses stated in this discussion may be the subject of further research.

## 6. Conclusions

This work studied the influence of 24 combinations of various factors on the intermetallic layer growth rate. These factors were the solder mask type, surface finish type, flux type, and the number of reflows. It is apparent that the flux type (its activity) and its amount in the joint during the reflow have a significant influence on the IML growth. Despite the identical amount of the flux in the solder paste before soldering, the solder mask influenced the amount of flux actually present in the joint during the reflow process and thus influenced the IML thickness. The use of a glossy mask resulted in a thinner IML.

The type of flux influenced the IML thickness even more significantly. The change of flux type (from ROL0 to ROL1) increased the thickness by up to 40%, which could negatively influence the mechanical properties of the solder joint [23]. The materials and their parameters (flux, surface finishes, solder alloy, solder mask, etc.) together with the parameters of the soldering process must be taken into consideration when predicting the intermetallic layers thickness.

## Figures and Tables

**Table 1:** Results of roughness measurement of the solder masks.

Solder Mask	Line Roughness R_a_ (µm)	Surface Roughness S_a_ (µm)
White	0.48 ± 0.13	0.52 ± 0.05
Black	1.05 ± 0.24	1.29 ± 0.14

## Data Availability

The data presented in this study are available on request from the corresponding author.
